# A conserved motif in JNK/p38-specific MAPK phosphatases as a determinant for JNK1 recognition and inactivation

**DOI:** 10.1038/ncomms10879

**Published:** 2016-03-18

**Authors:** Xin Liu, Chen-Song Zhang, Chang Lu, Sheng-Cai Lin, Jia-Wei Wu, Zhi-Xin Wang

**Affiliations:** 1 Key Laboratory of Ministry of Education for Protein Science, School of Life Sciences, Tsinghua University, Beijing 100084, China; 2 State Key Laboratory of Stress Cell Biology, School of Life Sciences, Xiamen University, Xiamen, Fujian 361005, China

## Abstract

Mitogen-activated protein kinases (MAPKs), important in a large array of signalling pathways, are tightly controlled by a cascade of protein kinases and by MAPK phosphatases (MKPs). MAPK signalling efficiency and specificity is modulated by protein–protein interactions between individual MAPKs and the docking motifs in cognate binding partners. Two types of docking interactions have been identified: D-motif-mediated interaction and FXF-docking interaction. Here we report the crystal structure of JNK1 bound to the catalytic domain of MKP7 at 2.4-Å resolution, providing high-resolution structural insight into the FXF-docking interaction. The ^285^FNFL^288^ segment in MKP7 directly binds to a hydrophobic site on JNK1 that is near the MAPK insertion and helix αG. Biochemical studies further reveal that this highly conserved structural motif is present in all members of the MKP family, and the interaction mode is universal and critical for the MKP-MAPK recognition and biological function.

## Abstract (web summary)

The important MAPK family of signalling proteins is controlled by MAPK phosphatases (MKPs). Here, the authors report the structure of MKP7 bound to JNK1 and characterise the conserved MKP-MAPK interaction.

## Graphical abstract

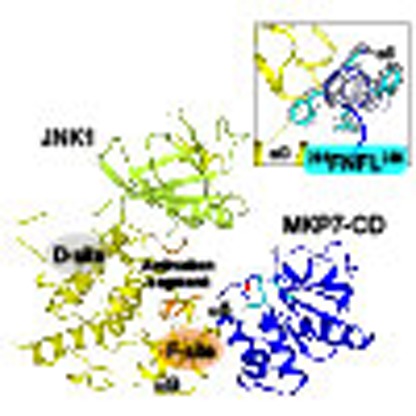

The mitogen-activated protein kinases (MAPKs) are central components of the signal-transduction pathways, which mediate the cellular response to a variety of extracellular stimuli, ranging from growth factors to environmental stresses^1^,^2^,^3^. The MAPK signalling pathways are evolutionally highly conserved. The basic assembly of MAPK pathways is a three-tier kinase module that establishes a sequential activation cascade: a MAPK kinase kinase activates a MAPK kinase, which in turn activates a MAPK. The three best-characterized MAPK signalling pathways are mediated by the kinases extracellular signal-regulated kinase (ERK), c-Jun N-terminal kinase (JNK) and p38. The ERK pathway is activated by various mitogens and phorbol esters, whereas the JNK and p38 pathways are stimulated mainly by environmental stress and inflammatory cytokines^4^,^5^,^6^. The MAPKs are activated by MAPK kinases that phosphorylate the MAPKs at conserved threonine and tyrosine residues within their activation loop. After activation, each MAPK phosphorylates a distinct set of protein substrates, which act as the critical effectors that enable cells to mount the appropriate responses to varied stimuli.

MAPKs lie at the bottom of conserved three-component phosphorylation cascades and utilize docking interactions to link module components and bind substrates^7^,^8^. Two types of docking motifs have been identified in MAPK substrates and cognate proteins: kinase-interacting motif (D-motif) and FXF-motif (also called DEF motif, docking site for ERK FXF). The best-studied docking interactions are those between MAP kinases and ‘D-motifs', which consists of two or more basic residues followed by a short linker and a cluster of hydrophobic residues. The D-motif-docking site (D-site) in MAPKs is situated in a noncatalytic region opposite of the kinase catalytic pocket and is comprised of a highly acidic patch and a hydrophobic groove. D-motifs are found in many MAPK-interacting proteins, including substrates, activating kinases and inactivating phosphatases, as well as scaffolding proteins. A second docking motif for MAPKs consists of two Phe residues separated by one residue (FXF-motif). This motif has been observed in several MAPK substrates^9^,^10^,^11^,^12^,^13^. The FXF-motif-binding site of ERK2 has been mapped to a hydrophobic pocket formed between the P+1 site, αG helix and the MAPK insert^14^. However, the generality and mechanism of the FXF-mediated interaction is unclear.

The physiological outcome of MAPK signalling depends on both the magnitude and the duration of kinase activation^15^. Downregulation of MAPK activity can be achieved through direct dephosphorylation of the phospho-threonine and/or tyrosine residues by various serine/threonine phosphatases, tyrosine phosphatases and dual-specificity phosphatases (DUSPs) termed MKPs. MKPs constitute a group of DUSPs that are characterized by their ability to dephosphorylate both phosphotyrosine and phosphoserine/phospho-threonine residues within a substrate^16^,^17^. Dysregulated expression of MKPs has been associated with pathogenesis of various diseases, and understanding their precise recognition mechanism presents an important challenge and opportunity for drug development^18^,^19^.

Here, we present the crystal structure of JNK1 in complex with the catalytic domain of MKP7. This structure reveals the molecular mechanism underlying the docking interaction between MKP7 and JNK1. In the JNK1–MKP7 complex, a hydrophobic motif (^285^FNFL^288^) that initiates the helix α5 in the MKP7 catalytic domain directly binds to the FXF-motif-binding site on JNK1, providing the structural insight into the classic FXF-type docking interaction. Biochemical and modelling studies further demonstrate that the molecular interactions mediate this key element for substrate recognition are highly conserved among all MKP-family members. Thus, our study reveals a hitherto unrecognized interaction mode for encoding complex target specificity among MAPK isoforms.

## Results

### Interaction of JNK1 with the MKP7 catalytic domain

**Figure 1:**
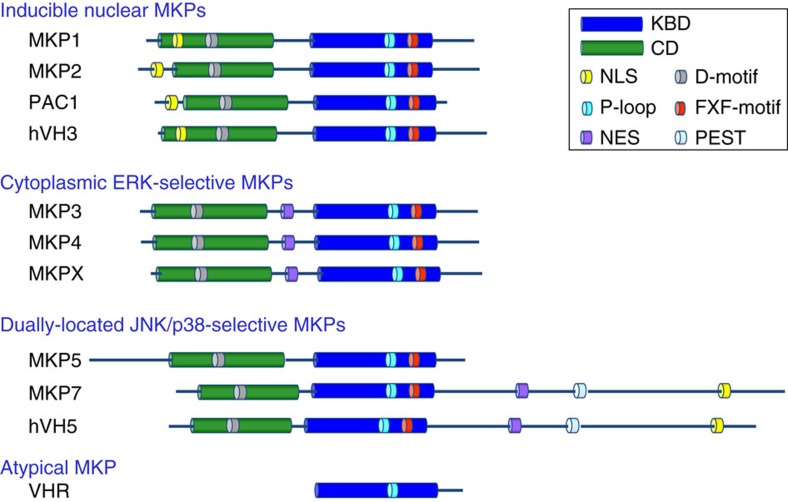
Domain structures of ten human MKPs and the atypical VHR. On the basis of sequence similarity, protein structure, substrate specificity and subcellular localization, the ten members of MKP family can be divided into three groups. The first subfamily comprises MKP1, MKP2, PAC1 and hVH3, which are inducible nuclear phosphatases and can dephosphorylate ERK (and JNK, p38) MAPKs. The second subfamily contains MKP3, MKP4 and MKPX, which are cytoplasmic ERK-specific MKPs. The third subfamily comprises MKP5, MKP7 and hVH5, which were located in both nucleus and cytoplasm, and selectively inactivate JNK and p38. All MKPs contain both the CD and KBD domains, whereas VHR, an atypical MKP, only contains a highly conserved catalytic domain. In addition to the CD and KBD, MKP7 contains a unique long C-terminal region that contains NES, NLS and PEST motifs, which has no effect on the binding ability and phosphatase activity of MKP7 toward MAPKs. NES, nuclear export signal; NLS, nuclear localization signal; PEST, C-terminal sequence rich in prolines, glutamates, serines and threonines.

DUSPs belong to the protein-tyrosine phosphatases (PTPase) superfamily, which is defined by the PTPase-signature motif CXXGXXR^20^. MKPs represent a distinct subfamily within a larger group of DUSPs. In mammalian cells, the MKP subfamily includes 10 distinct catalytically active MKPs. All MKPs contain a highly conserved C-terminal catalytic domain (CD) and an N-terminal kinase-binding domain (KBD)^15^,^21^. The KBD is homologous to the rhodanese family and contains an intervening cluster of basic amino acids, which has been suggested to be important for interacting with the target MAPKs. On the basis of sequence similarity, substrate specificity and predominant subcellular localization, the MKP family can be further divided into three groups (Fig. 1). Biochemical and structural studies have revealed that the KBD of MKPs is critical for MKP3 docking to ERK2, and MKP5 binding to p38α, although their binding mechanisms are completely different^22^,^23^. However, it is unknown if other MAPKs can interact with the KBD of their cognate phosphatases in the same manner as observed for recognition of ERK2 and p38α by their MKPs, or whether they recognize distinct docking motifs of MKPs.

**Figure 2:**
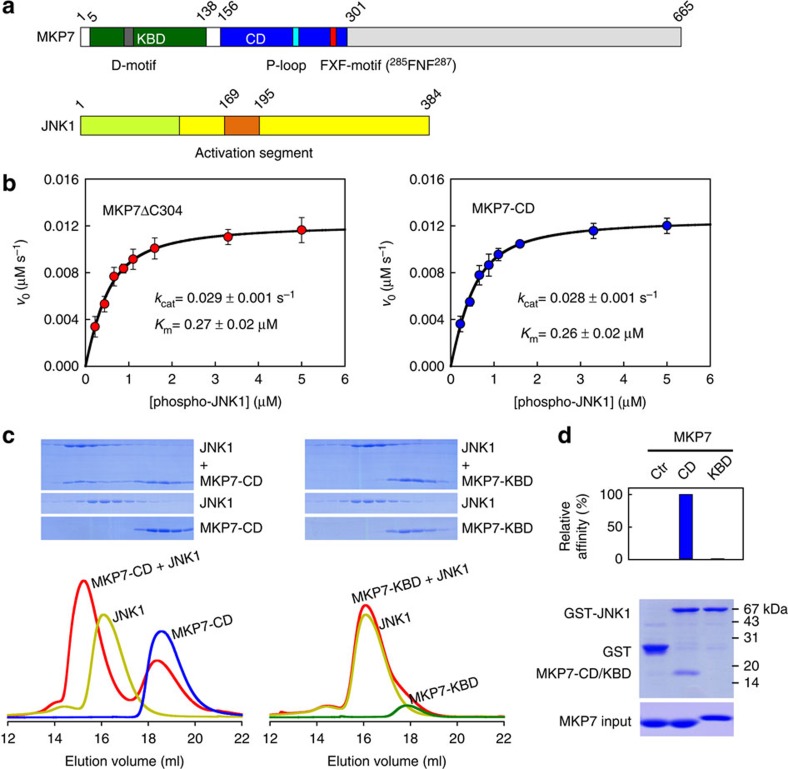
MKP7-CD is crucial for JNK1 binding and enzyme catalysis. (**a**) Domain organization of human MKP7 and JNK1. The KBD and CD of MKP7 are shown in green and blue, and the N-lobe and C-lobe of JNK1 are coloured in lemon and yellow, respectively. The key structural elements are indicated. The colour scheme is the same in the following figures unless indicated otherwise. (**b**) Plots of initial velocity of the MKP7-catalysed reaction versus phospho-JNK1 concentration. The solid lines are best-fitting results according to equation (1). Each experiment was performed in replicate for at least three times. The error bars represent s.e.m. (**c**) Gel filtration analysis for interaction of JNK1 with MKP7-CD and MKP7-KBD. (**d**) GST-mediated pull-down assay for interaction of JNK1 with MKP7-CD and MKP7-KBD. The top panel shows the relative affinities of MKP7-CD and MKP7-KBD to JNK1, with the affinity of MKP7-CD defined as 100%; the middle panel is the electrophoretic pattern of MKP7 and JNK1 after GST pull-down assays. The protein amounts of MKP7 used are shown at the bottom.

MKP7, the biggest molecule in the MKP family, selectively inactivates JNK and p38 following stress activation^24^. In addition to the CD and KBD, MKP7 has a long C-terminal region that contains both nuclear localization and export sequences by which MKP7 shuttles between the nucleus and the cytoplasm (Fig. 2a). To quantitatively assess the contribution of the N-terminal domain to the MKP7-catalysed JNK1 dephosphorylation, we first measured the kinetic parameters of the C-terminal truncation of MKP7 (MKP7ΔC304, residues 5–303) and MKP7-CD (residues 156–301) towards phosphorylated JNK1 (pJNK1). Figure 2b shows the variation of initial rates of the MKP7ΔC304 and MKP7-CD-catalysed reaction with the concentration of phospho-JNK1. Because the concentrations of MKP7 and pJNK1 were comparable in the reaction, the assumption that the free-substrate concentration is equal to the total substrate concentration is not valid. Thus, the kinetic data were analysed using the general initial velocity equation, taking substrate depletion into account:


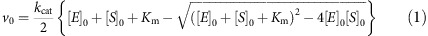


The *k*_cat_ and *K*_m_ of the MKP7-CD (0.028 s^−1^ and 0.26 μM) so determined were nearly identical to those of MKP7ΔC304 (0.029 s^−1^ and 0.27 μM), indicating that the MKP7-KBD has no effect on enzyme catalysis.

We next examined the interaction of JNK1 with the CD and KBD of MKP7 by gel filtration analysis. When 3 molar equivalents of CD were mixed with 1 molar equivalent of JNK1, a significant amount of CD co-migrated with JNK1 to earlier fractions, and the excess amount of CD was eluted from the size exclusion column as a monomer, indicating stable complex formation (Fig. 2c). In contrast, no KBD–JNK1 complex was detected when 3 molar equivalents of KBD were mixed with 1 molar equivalent of JNK1. To further confirm the JNK1–MKP7-CD interaction, we performed a pull-down assay using the purified proteins. As shown in Fig. 2d, the CD of MKP7 can be pulled down by JNK1, while the KBD failed to bind to the counterpart protein. Taken together, our data indicate that the CD of MKP7, but not the KBD domain, is responsible for JNK substrate-binding and enzymatic specificity.

### Crystal structure of JNK1 in complex with the MKP7-CD

**Figure 3:**
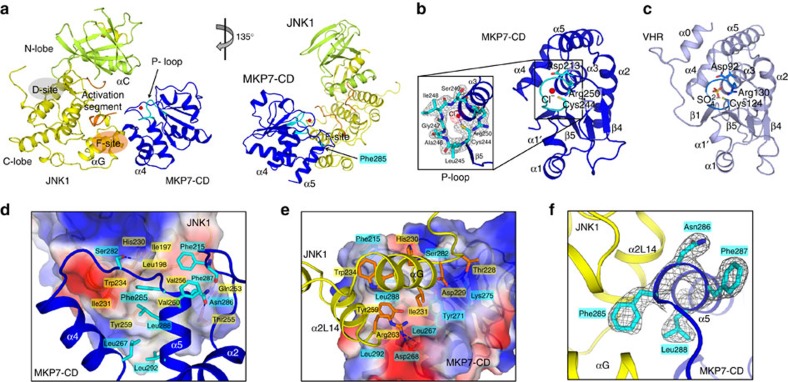
Structure of JNK1 in complex with MKP7-CD. (**a**) Ribbon diagram of JNK1–MKP7-CD complex in two views related by a 45° rotation around a vertical axis. (**b**) Structure of MKP7-CD with its active site highlight in cyan. The 2*F*_o_−*F*_c_ omit map (contoured at 1.5σ) for the P-loop of MKP7-CD is shown at inset of **b**. (**c**) Structure of VHR with its active site highlighted in marine blue. (**d**) Close-up view of the JNK1–MKP7 interface showing interacting amino acids of JNK1 (orange) and MKP7-CD (cyan). The JNK1 is shown in surface representation coloured according to electrostatic potential (positive, blue; negative, red). (**e**) Interaction networks mainly involving helices α4 and α5 from MKP7-CD, and αG and α2L14 of JNK1. MKP7-CD is shown in surface representation coloured according to electrostatic potential (positive, blue; negative, red). Blue dashed lines represent polar interactions. (**f**) The 2*F*_o_−*F*_c_ omit map (contoured at 1.5σ) clearly shows electron density for the ^285^FNFL^288^ segment of MKP7-CD.

To understand the molecular basis of JNK1 recognition by MKP7, we determined the crystal structure of unphosphorylated JNK1 in complex with the MKP7-CD (Fig. 3a, Supplementary Fig. 1a and Table 1). In the complex, JNK1 has its characteristic bilobal structure comprising an N-terminal lobe rich in β-sheet and a C-terminal lobe that is mostly α-helical. The overall folding of MKP7-CD is typical of DUSPs, with a central twisted five-stranded β-sheet surrounded by six α-helices. One side of the β-sheet is covered with two α-helices and the other is covered with four α-helices (Fig. 3b). The catalytic domain of MKP7 interacts with JNK1 through a contiguous surface area that is remote from the active site. MKP7-CD is positioned onto the JNK1 molecule so that the active site of the phosphatase faces towards the activation segment. In an alignment of the structure of MKP7-CD with that of VHR^25^, an atypical ‘MKP' consisting of only a catalytic domain, 119 of 147 MKP7-CD residues could be superimposed with a r.m.s.d. (root mean squared deviation) of 1.05 Å (Fig. 3c). The most striking difference is that helix α0 and loop α0–β1 of VHR are absent in MKP7-CD. Another region that cannot be aligned with VHR is found in loop β3–β4. This loop is shortened by nine residues in MKP7-CD compared with that in VHR. Since helix α0 and the following loop α0–β1 are known for a substrate-recognition motif of VHR and other phosphatases, the absence of these moieties implicates a different substrate-binding mode of MKP7.

The active site of MKP7 consists of the phosphate-binding loop (P-loop, Cys244-Leu245-Ala246-Gly247-Ile248-Ser249-Arg250), and Asp213 in the general acid loop (Fig. 3b and Supplementary Fig. 1b). The MKP7-CD structure near the active site exhibits a typical active conformation as found in VHR and other PTPs^25^. The catalytic residue, Cys244, is located just after strand β5 and optimally positioned for nucleophilic attack^26^,^27^,^28^. Asp213 in MKP7 also adopts a position similar to that of Asp92 in VHR (Supplementary Fig. 1c), indicating that Asp213 is likely to function as the general acid in MKP7. We also observed the binding of a chloride ion in the active site of MKP7-CD. It is located 3.36 Å from the Cys244 side chain and makes electrostatic interactions with the dipole moment of helix α3 and with several main-chain amide groups. The side chain of strictly conserved Arg250 is oriented towards the negatively charged chloride, similar to the canonical phosphate-coordinating conformation. Thus this chloride ion is a mimic for the phosphate group of the substrate, as revealed by a comparison with the structure of PTP1B in complex with phosphotyrosine^29^ (Supplementary Fig. 1d). Although the catalytically important residues in MKP7-CD are well aligned with those in VHR, the residues in the P-loop of MKP7 are smaller and have a more hydrophobic character than those of VHR (Cys124-Arg125-Glu126-Gly127-Tyr128-Gly129-Arg130; Fig. 3b,c). The difference in the polarity/hydrophobicity of the surface may also point to the origin of the differences in the substrate-recognition mechanism for these two phosphatases (Supplementary Fig. 1e,f).

In the complex, MKP7-CD and JNK1 form extensive protein–protein interactions involving the C-terminal helices of MKP7-CD and C-lobe of JNK1 (Fig. 3d,e). As a result, the buried solvent-accessible surface area is ∼1,315 Å. In the C-terminal domain, JNK1 has an insertion after the helix αG. This insertion consists of two helices (α1L14 and α2L14) that are common to all members of the MAPK family. The interactive surface in JNK1, formed by the helices αG and α2L14, displays a hydrophobic region, centred at Trp234 (Fig. 3d). The MKP7-docking region includes two helices, α4 and α5, and the general acid loop. The aromatic ring of Phe285 on MKP7 α5-helix is nestled in a hydrophobic pocket on JNK1, formed by side chains of Ile197, Leu198, Ile231, Trp234, Val256, Tyr259, Val260 and the aliphatic portion of His230 (Fig. 3d,f and Supplementary Fig. 1g). In addition, there are hydrogen bonds between Ser282 and Asn286 of MKP7 and His230 and Thr255 of JNK1, and the main chain of Phe215 in the general acid loop of MKP7 is hydrogen-bonded to the side chain of Gln253 in JNK1. The second interactive area involves the α4 helix of MKP7 and charged/polar residues of JNK1 (Fig. 3e). The carboxylate of Asp268 in MKP7 forms a salt bridge with side chain of Arg263 in JNK1, and Lys275 of MKP7 forms a hydrogen bond and a salt bridge with Thr228 and Asp229 of JNK1, respectively.

### Mutational analysis of the JNK1–MKP7 docking interface

**Figure 4:**
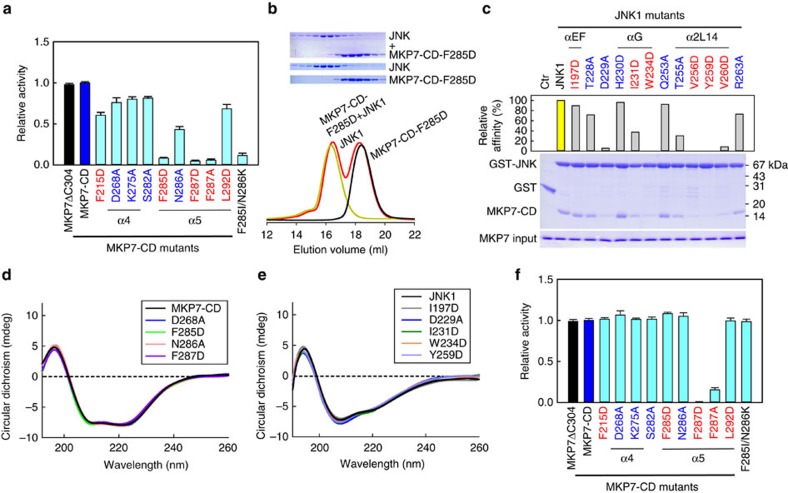
Mutational analysis on interactions between MKP7-CD and JNK1. (**a**) Effects of mutations in MKP7-CD on the JNK1 dephosphorylation (mean±s.e.m., *n*=3). Residues involved in hydrophobic and hydrophilic contacts are coloured in red and blue, respectively. (**b**) Gel filtration analysis for interaction of JNK1 with MKP7-CD mutant F285D. Mutant F285D and JNK1 were eluted as monomers, with the molecular masses of ∼17 and 44 kDa, respectively. However, in contrast to the wild-type MKP7-CD, mutant F285D did not co-migrate with JNK1. (**c**) Pull-down assays of MKP7-CD by GST-tagged JNK1 mutants. The top panel shows the relative affinities of MKP7-CD to JNK1 mutants, with the affinity of wild-type JNK1 defined as 100%, the middle panel is the electrophoretic pattern of MKP7-CD and JNK1 mutants after GST pull-down assays. The protein amounts of MKP7-CD used are shown at the bottom. (**d**) Circular dichroism spectra for MKP7-CD wild type and mutants. Measurements were averaged for three scans. (**e**) Circular dichroism spectra for JNK1 wild type and mutants. Measurements were averaged for three scans. (**f**) Effects of mutations in MKP7-CD on the *p*NPP hydrolysis reaction (mean±s.e.m., *n*=3).

To assess the importance of the aforementioned interactions, we generated a series of point mutations on the MKP7-CD and examined their effect on the MKP7-catalysed JNK1 dephosphorylation (Fig. 4a). When the hydrophobic residues Phe285 and Phe287 on the α5 of MKP7-CD were replaced by Asp or Ala, their phosphatase activities for JNK1 dephosphorylation decreased ∼10-fold. In comparison, replacement of the other residues (Phe215, Asp268, Lys275, Ser282, Asn286 and Leu292) with an Ala or Asp individually led to a modest decrease in catalytic efficiencies, suggesting that this position may only affect some selectivity of MKP. Mutation of Leu288 markedly reduced its solubility when expressed in *Escherichia coli*, resulting in the insoluble aggregation of the mutant protein. Gel filtration analysis further confirmed the key role of Phe285 in the MKP7–JNK1 interaction: no F285D–JNK1 complex was detected when 3 molar equivalents of MKP7-CD (F285D) were mixed with 1 molar equivalent of JNK1 (Fig. 4b). Interestingly, mutation of Phe287 results in a considerable loss of activity against pJNK1 without altering the affinity of MKP7-CD for JNK1 (Supplementary Fig. 2a). We also generated a series of point mutations in the JNK1 and assessed the effect on JNK1 binding using the GST pull-down assay (Fig. 4c). Substitution at Asp229, Trp234, Thr255, Val256, Tyr259 and Val260 significantly reduced the binding affinity of MKP7-CD for JNK. To determine whether the deficiencies in their abilities to bind partner proteins or carry out catalytic function are owing to misfolding of the purified mutant proteins, we also examined the folding properties of the JNK1 and MKP7 mutants with circular dichroism. The spectra of these mutants are similar to the wild-type proteins, indicating that these mutants fold as well as the wild-type proteins (Fig. 4d,e). Taken together, these results are consistent with the present crystallographic model, which reveal the hydrophobic contacts between the MKP7 catalytic domain and JNK1 have a predominant role in the enzyme–substrate interaction, and hydrophobic residue Phe285 in the MKP7-CD is a key residue for its high-affinity binding to JNK1.

It has previously been reported that several cytosolic and inducible nuclear MKPs undergo catalytic activation upon interaction with the MAPK substrates^15^. This allosteric activation of MKP3 has been well-documented *in vitro* using *p*NPP, a small-molecule phosphotyrosine analogue of its normal substrate^30^,^31^. We then assayed *p*NPPase activities of MKP7ΔC304 and MKP7-CD in the presence of JNK1. Incubation of MKP7 with JNK1 did not markedly stimulate the phosphatase activity, which is consistent with previous results that MKP7 solely possesses the intrinsic activity (Supplementary Fig. 2b). The small *p*NPP molecule binds directly at the enzyme active site and can be used to probe the reaction mechanism of protein phosphatases. We therefore examined the effects of the MKP7-CD mutants on their *p*NPPase activities. As shown in Fig. 4f, all the mutants, except F287D/A, showed little or no activity change compared with the wild-type MKP7-CD. In the JNK1/MKP7-CD complex structure, Phe287 of MKP7 does not make contacts with JNK1 substrate. It penetrates into a pocket formed by residues from the P-loop and general acid loop and forms hydrophobic contacts with the aliphatic portions of side chains of Arg250, Glu217 and Ile219, suggesting that Phe287 in MKP7 would play a similar role to that of its structural counterpart in the PTPs (Gln266 in PTP1B) and VHR (Phe166 in VHR) in the precise alignment of active-site residues in MKP7 with respect to the substrate for efficient catalysis^32^,^33^,^34^,^35^ (Supplementary Fig. 2c).

**Figure 5:**
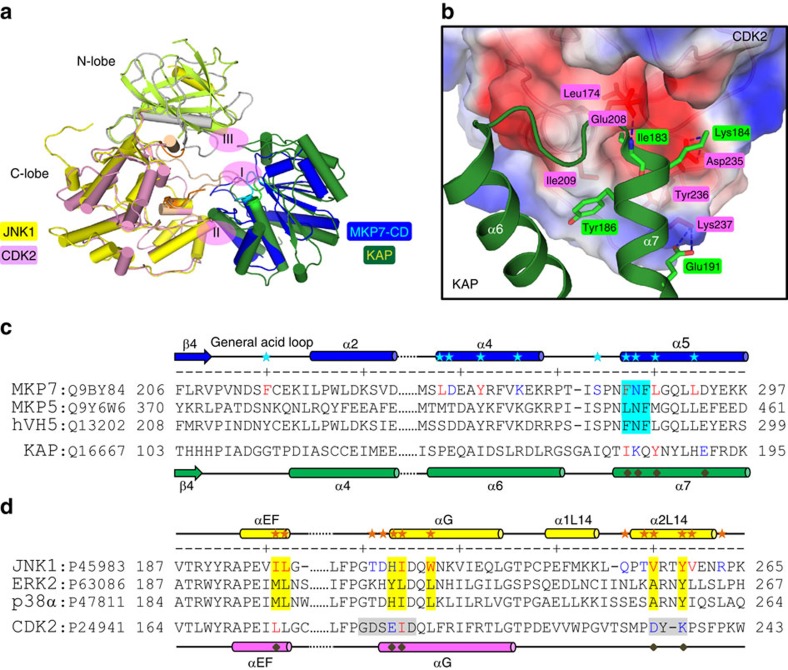
Comparison of CDK2-KAP and JNK1–MKP7-CD. (**a**) Superposition of the complex structures of CDK2-KAP (PDB 1FQ1) and JNK1–MKP7-CD. The N-lobe and C-lobe of CDK2 are coloured in grey and pink, respectively, and KAP is coloured in green. The interactions between these two proteins consist of three discontinuous contact regions, centred at the multiple hydrogen bonds between the pThr160 of CDK2 and the active site of KAP (region I). Interestingly, the recognition of CDK2 by KAP is augmented by a similar interface as that observed in the complex of JNK1 and MKP7-CD (region II). (**b**) Interactions networks at the auxiliary region II mainly involving helix α7 from KAP and the αG helix and following L14 loop of CDK2. The orientation of the panel is almost identical to those of Fig. 3d. The CDK2 is shown in surface representation coloured according to the electrostatic potential (positive, blue; negative, red). Residues of KAP and CDK2 are highlighted as green and red sticks, respectively. Blue dashed lines represent polar interactions. One remarkable difference between these two kinase-phosphatase complexes is that helix α6 of KAP (corresponding to helix α4 of MKP7-CD) plays little, if any, role in the formation of a stable heterodimer of CDK2 and KAP. (**c**) Sequence alignment of the JNK-interacting regions on MKPs. Residues of MKP7-CD involved in JNK1 recognition are indicated by cyan asterisks, and the conserved FXF-motif is highlighted in cyan. The secondary structure assignments of MKP7-CD and KAP are shown above and below each sequence. (**d**) Sequence alignment of the F-site regions on MAPKs. Residues of JNK1 involved in recognition of MKP7 are indicated by orange asterisks, and those forming the F-site are highlighted in yellow.

Kinase-associated phosphatase (KAP), a member of the DUSP family, plays a crucial role in cell cycle regulation by dephosphorylating the pThr160 residue of CDK2 (cyclin-dependent kinase 2). The crystal structure of the CDK2/KAP complex has been determined at 3.0 Å (Fig. 5a)^36^. The interface between these two proteins consists of three discontinuous contact regions. Biochemical results suggested that the affinity and specificity between KAP and CDK2 results from the recognition site comprising CDK2 residues from the αG helix and L14 loop and the N-terminal helical region of KAP (Fig. 5b). There is a hydrogen bond between the main-chain nitrogen of Ile183 (KAP) and side chain oxygen of Glu208 (CDK2), and salt bridges between Lys184 of KAP and Asp235 of CDK2. Structural analysis and sequence alignment reveal that one of the few differences between MKP7-CD and KAP in the substrate-binding region is the presence of the motif FNFL in MKP7-CD, which corresponds to IKQY in KAP (Fig. 5c). The substitution of the two hydrophobic residues with charged/polar residues (F285I/N286K) seriously disrupts the hydrophobic interaction required for MKP7 binding on JNK1 (Fig. 4a). In addition, His230 and Val256 in JNK1 are replaced by the negatively charged residues Glu208 and Asp235 in CDK2 (Fig. 5d), and the charge distribution on the CDK2 interactive surface is quite different from that of JNK. These data indicated that a unique hydrophobic pocket formed between the MAPK insert and αG helix plays a major role in the substrate recognition by MKPs.

### F-site interaction is crucial for JNK1 inactivation *in vivo*

**Figure 6:**
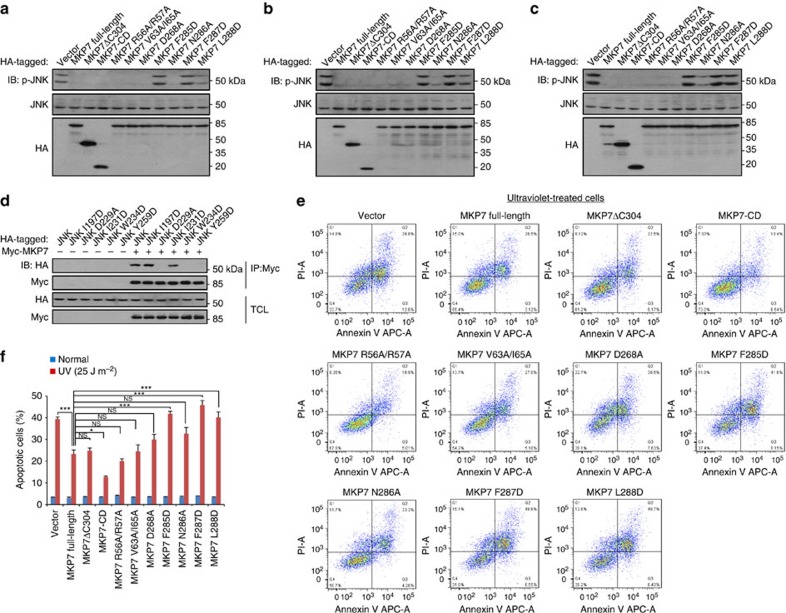
FXF-motif is critical for controlling the phosphorylation of JNK and ultraviolet-induced apoptosis. (**a**–**c**) FXF-motif is essential for the dephosphorylation of JNK by MKP7. HEK293T cells were infected with lentiviruses expressing MKP7 and its mutants (1.0 μg). After 36 h infection, cells were untreated in **a**, stimulated with 30 μM etoposide for 3 h in **b** or irradiated with 25 J m^−2^ ultraviolet light at 30 min before lysis in **c**. Whole-cell extracts were then immunoblotted with antibody indicated. Shown is a typical immunoblot for phosphorylated JNK from three independent experiments. (**d**) F-site is required for JNK1 to interact with MKP7. HEK293T cells were co-transfected with MKP7 full-length (1.0 μg) and JNK1 (wild type or mutants as indicated, 1.0 μg). At 16 h post transfection, cells were lysed. Whole-cell extracts were then immunoprecipitated with antibody against Myc for MKP7; immunobloting was carried out with antibodies indicated. IP, immunoprecipitation; TCL, total cell lysate. Shown is a typical result from three independent experiments. (**e**) Effect of MKP7 (wild type or mutants) expression on ultraviolet-induced apoptosis. HeLa cells were infected with lentiviruses expressing MKP7 full-length and its mutants. At 36 h post infection, cells were irradiated with 25 J m^−2^ ultraviolet light and collected at 6 h after irradiation. Cells were then subjected to flow cytometry analysis. Apoptotic cells were determined by Annexin-V-APC/PI staining. The results using Annexin-V stain for membrane phosphatidylserine eversion, combined with propidium iodide (PI) uptake to evaluate cells whose membranes had been compromised. Staining with both Annexin-V and PI indicate apoptosis (upper right quadrant). The values shown in the lower left, and upper right quadrants of each panel represent the percentage of viable, and apoptotic cells, respectively. All results are representative of three independent experiments. (**f**) Statistical analysis of apoptotic cells (mean±s.e.m., *n*=3), *P<0.05, ***P<0.001 (ANOVA followed by Tukey's test). NS, not significant.

JNK is activated following cellular exposure to a number of acute stimuli such as anisomycin, H_2_O_2_, ultraviolet light, sorbitol, DNA-damaging agents and several strong apoptosis inducers (etoposide, cisplatin and taxol)^37^,^38^,^39^. To assess the effects of MKP7 and its mutants on the activation of endogenous JNK *in vivo*, HEK293T cells were transfected with blank vector or with HA-tagged constructs for full-length MKP7, MKP7ΔC304 and MKP7-CD or MKP7 mutants, and stimulated with ultraviolet or etoposide treatment. As shown in Fig. 6a–c, immunobloting showed similar expression levels for the different MKP7 constructs in all the cells. Overexpressed full-length MKP7, MKP7ΔC304 and MKP7-CD significantly reduced the endogenous level of phosphorylated JNK compared with vector-transfected cells. Parallel experiments showed clearly that the D-motif mutants (R56A/R57A and V63A/I65A) dephosphorylated JNK as did the wild type under the same conditions, further confirming that the MKP7-KBD is not required for the JNK inactivation *in vivo*. Consistent with the *in vitro* data, the level of phosphorylated JNK was not or little altered in MKP7 FXF-motif mutants (F285D, F287D and L288D)-transfected cells, and the MKP7 D268A and N286A mutants retained the ability to reduce the phosphorylation levels of JNK. We next tested *in vivo* interactions between JNK1 mutants and full-length MKP7 by coimmunoprecipitation experiments under unstimulated conditions. When co-expressed in HEK293T cells, wild-type (HA)-JNK1 was readily precipitated with (Myc)-MKP7 (Fig. 6d), indicating that MKP7 binds dephosphorylated JNK1 protein *in vivo*. In agreement with the *in vitro* pull-down results, the mutants D229A, W234D and Y259D were not co-precipitated with MKP7, and the I231D mutant had only little effect on the JNK1–MKP7 interaction (Fig. 6d and Supplementary Fig. 3a).

Activation of the JNK signalling pathway is frequently associated with apoptotic cell death, and inhibition of JNK can prevent apoptotic death of multiple cells^6^,^40^,^41^,^42^,^43^. To examine whether the inhibition of JNK activity by MKP7 would provide protections against the apoptosis, we analysed the rate of apoptosis in ultraviolet-irradiated cells transfected with MKP7 (wild type or mutants) by flow cytometry. The results showed similar apoptotic rates between cells transfected with blank vector or with MKP7 (wild type or mutants) under unstimulated conditions (Supplementary Fig. 3b), while ultraviolet-irradiation significantly increased apoptotic rate in cells transfected with blank vector (Fig. 6e). Expressions of wild-type MKP7, MKP7ΔC304 and MKP7-CD significantly decreased the proportion of apoptotic cells after ultraviolet treatment. Moreover, treatment of cells expressing MKP7-KBD mutants (R56A/R57A and V63A/I65A) decreased the apoptosis rates to a similar extent as MKP7 wild type did. In contrast, cells transfected with the MKP7 FXF-motif mutants (F285D, F287D and L288D) showed little protective effect after ultraviolet treatment and similar levels of apoptosis rates were detected to cells transfected with control vectors (Fig. 6e,f). Taken together, our results suggested that FXF-motif-mediated, rather than KBD-mediated, interaction is essential for MKP7 to block ultraviolet-induced apoptosis.

### A similar docking mechanism for JNK1 recognition by MKP5

**Figure 7:**
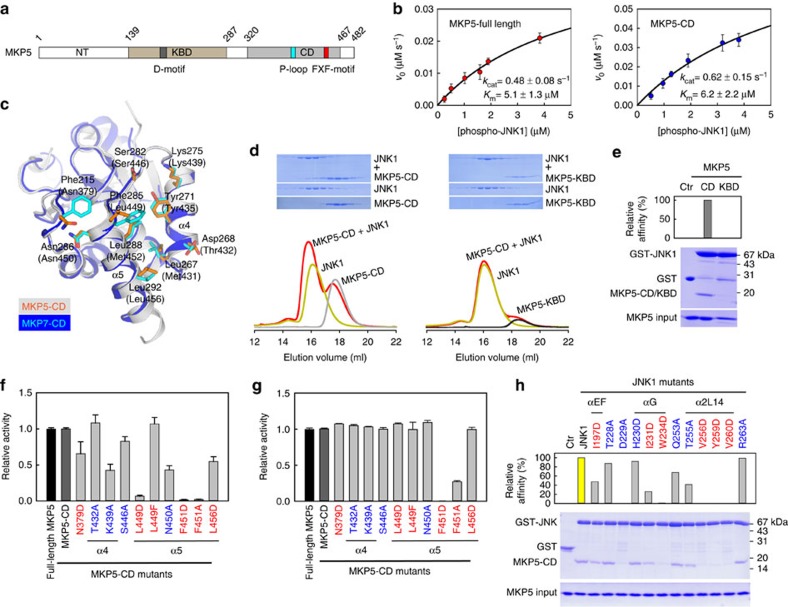
MKP5-CD is crucial for JNK1 binding and enzyme catalysis. (**a**) Domain organization of human MKP5. The KBD and CD of MKP5 are shown in brown and grey, respectively. (**b**) Plots of initial velocity of the MKP5-catalysed reaction versus phospho-JNK1 concentration. The solid lines are best-fitting results according to the Michaelis–Menten equation with *K*_m_ and *k*_cat_ values indicated. Each experiment was performed in replicate for at least three times. The error bars represent s.e.m. (**c**) Structural comparison of the JNK-interacting residues on MKP5-CD (PDB 1ZZW) and MKP7-CD. The corresponding residues on MKP5 are depicted as orange sticks, and MKP5 residues numbers are in parentheses. (**d**) Gel filtration analysis for interaction of JNK1 with MKP5-CD and MKP5-KBD. (**e**) GST-mediated pull-down assays for interaction of JNK1 with MKP5-CD and MKP5-KBD. The panels are arranged the same as in Fig. 2d. (**f**) Effects of mutations in MKP5-CD on the JNK1 dephosphorylation (mean±s.e.m., *n*=3). (**g**) Effects of mutations in MKP5-CD on the *p*NPP hydrolysis reaction (mean±s.e.m., *n*=3). (**h**) Pull-down assays of MKP5-CD by GST-tagged JNK1 mutants. The panels are arranged the same as in Fig. 4c.

MKP5 belongs to the same subfamily as MKP7. MKP5 is unique among the MKPs in possessing an additional domain of unknown function at the N-terminus^44^ (Fig. 7a). The KBD of MKP5 interacts with the D-site of p38α to mediate the enzyme–substrate interaction. Deletion of the KBD in MKP5 leads to a 280-fold increase in *K*_m_ for p38α substrate^23^. In contrast to p38α substrate, deletion of the MKP5-KBD had little effects on the kinetic parameters for the JNK1 dephosphorylation, indicating that the KBD of MKP5 is not required for the JNK1 dephosphorylation (Fig. 7b). The substrate specificity constant *k*_cat_ /*K*_m_ value for MKP5-CD was calculated as 1.0 × 10^5^ M^−1^ s^−1^, which is very close to that of MKP7-CD (1.07 × 10^5^ M^−1^ s^−1^). The crystal structure of human MKP5-CD has been determined^45^. Comparisons between catalytic domains structures of MKP5 and MKP7 reveal that the overall folds of the two proteins are highly similar, with only a few regions exhibiting small deviations (r.m.s.d. of 0.79 Å; Fig. 7c).

Given the distinct interaction mode revealed by the crystal structure of JNK1–MKP7-CD, one obvious question is whether this is a general mechanism used by all members of the JNK-specific MKPs. To address this issue, we first examined the docking ability of JNK1 to the KBD and CD of MKP5 using gel filtration analysis and pull-down assays. It can be seen from gel filtration experiments that JNK1 can forms a stable heterodimer with MKP5-CD in solution, but no detectable interaction was found with the KBD domain (Fig. 7d). Pull-down assays also confirmed the protein–protein interactions observed above. The catalytic domain of MKP5, but not its KBD, was able to pull-down a detectable amount of JNK1 (Fig. 7e), implicating a different substrate-recognition mechanisms for p38 and JNK MAPKs. To further test our hypothesis, we generated forms of MKP5-CD bearing mutations corresponding to the changes we made on MKP7-CD on the basis of sequence and structural alignment and examined their effects on the phosphatase activity. As shown in Fig. 7f, the T432A and L449F MKP5 mutant showed little or no difference in phosphatase activity, whereas the other mutants showed reduced specific activities of MKP5. As in the case of MKP7, all the mutants, except F451D/A, showed no *p*NPPase activity changes compared with the wild-type MKP5-CD (Fig. 7g), and the point mutations in JNK1 also reduced the binding affinity of MKP5-CD for JNK1 (Fig. 7h). In addition, there were no significant differences in the CD spectra between wild-type and mutant proteins, indicating that the overall structures of these mutants did not change significantly from that of wild-type MKP5 protein (Supplementary Fig. 4a). Taken together, our results suggest that MKP5 binds JNK1 in a docking mode similar to that in the JNK1–MKP7 complex, and the detailed interaction model can be generated using molecular dynamics simulation based on the structure of JNK1–MKP7-CD complex (Supplementary Fig. 4b,c). In this model, the MKP5-CD adopts a conformation nearly identical to that in its unbound form, suggesting that the conformation of the catalytic domain undergoes little change, if any at all, upon JNK1 binding. In particular, Leu449 of MKP5, which is equivalent to the key residue Phe285 of MKP7, buried deeply within the hydrophobic pocket of JNK1 in the same way as Phe285 in the JNK1–MKP7-CD complex (Supplementary Fig. 4d). Despite the strong similarities between JNK1–MKP5-CD and JNK1–MKP7-CD, however, there are differences. The JNK1–MKP7-CD interaction is better and more extensive. Asp268 of MKP7-CD forms salt bridge with JNK1 Arg263, whereas the corresponding residue Thr432 in MKP5-CD may not interact with JNK1. In addition, the key interacting residues of MKP7-CD, Phe215, Leu267 and Leu288, are replaced by less hydrophobic residues, Asn379, Met431 and Met452 in MKP5-CD (Fig. 5c), respectively, which may result in weaker hydrophobic interactions between MKP5-CD and JNK1. This is consistent with the experimental observation showing that JNK1 binds to MKP7-CD much more tightly than MKP5-CD (*K*_m_ value of MKP5-CD for pJNK1 substrate is ∼20-fold higher than that of MKP7-CD).

## Discussion

The MAPKs p38, ERK and JNK, are central to evolutionarily conserved signalling pathways that are present in all eukaryotic cells. Each MAPK cascade is activated in response to a diverse array of extracellular signals and culminates in the dual-phosphorylation of a threonine and a tyrosine residue in the MAPK-activation loop^2^. The propagation of MAPK signals is attenuated through the actions of the MKPs. Most studies have focused on the dephosphorylation of MAPKs by phosphatases containing the ‘kinase-interaction motif ' (D-motif), including a group of DUSPs (MKPs) and a distinct subfamily of tyrosine phosphatases (HePTP, STEP and PTP-SL)^46^,^47^. Crystal structures of ERK2 bound with the D-motif sequences derived from MKP3 and HePTP have been reported^22^,^48^. These structures revealed that linear docking motifs in interacting proteins bind to a common docking site on MAPKs outside the kinase active site. The particular amino acids and their spacing within D-motif sequences and amino acid composition of the docking sites on MAPKs appear to determine the specificity of D-motifs for individual MAPKs.

Recently, the crystal structure of a complex between the KBD of MKP5 and p38α has been obtained^23^. This complex has revealed a distinct interaction mode for MKP5. The KBD of MKP5 binds to p38α in the opposite polypeptide direction compared with how the D-motif of MKP3 binds to ERK2. In contrast to the canonical D-motif-binding mode, separate helices, α2 and α3′, in the KBD of MKP5 engage the p38α-docking site. Further structural and biochemical studies indicate that KBD of MKP7 may interact with p38α in a similar manner to that of MKP5. In contrast to MKP5, removal of the KBD domain from MKP7 does not drastically affect enzyme catalysis, and the kinetic parameters of MKP7-CD for p38α substrate are very similar to those for JNK1 substrate^23^. Taken together, these results suggest that MKP7 utilizes a bipartite recognition mechanism to achieve the efficiency and fidelity of p38α signalling. The MKP7-KBD docks to the D-site located on the back side of the p38α catalytic pocket for high-affinity association, whereas the interaction of the MKP7-CD with another p38α structural region, which is close to the activation loop, may not only stabilize binding but also provide contacts crucial for organizing the MKP7 active site with respect to the phosphoreceptor in the p38α substrate for efficient dephosphorylation.

In addition to the canonical D-site, the MAPK ERK2 contains a second binding site utilized by transcription factor substrates and phosphatases, the FXF-motif-binding site (also called F-site), that is exposed in active ERK2 and the D-motif peptide-induced conformation of MAPKs^9^,^10^,^49^. This hydrophobic site was first identified by changes in deuterium exchange profiles, and is near the MAPK insertion and helix αG. Interestingly, many of the equivalent residues in JNK1, important for MKP7-CD recognition, are also used for substrate binding by ERK2 (ref. 14), indicating that this site is overlapped with the DEF-site previously identified in ERK2 (Fig. 5d). MKP3 is highly specific in dephosphorylating and inactivating ERK2, and the phosphatase activity of the MKP3-catalysed *p*NPP reaction can be markedly increased in the presence of ERK2 (refs 30, 31). Sequence alignment of all MKPs reveals a high degree of conservation of residues surrounding the interacting region observed in JNK1–MKP7-CD complex (Supplementary Fig. 5). Therefore, it is tempting to speculate that the catalytic domain of MKP3 may bind to ERK2 in a manner analogous to the way by which MKP7-CD binds to JNK1. A comprehensive examination of the molecular basis of the specific ERK2 recognition by MKP3 is underway. The ongoing work demonstrates that although the overall interaction modes are similar between the JNK1–MKP7-CD and ERK2–MKP3-CD complexes, the ERK2–MKP3-CD interaction is less extensive and helix α4 from MKP3-CD does not interact directly with ERK2. The FXF-motif-mediated interaction is critical for both pERK2 inactivation and ERK2-induced MKP3 activation (manuscript in preparation).

In summary, we have resolved the structure of JNK1 in complex with the catalytic domain of MKP7. This structure reveals an FXF-docking interaction mode between MAPK and MKP. Results from biochemical characterization of the Phe285 and Phe287 MKP7 mutants combined with structural information support the conclusion that the two Phe residues serve different roles in the catalytic reaction. Phe285 is essential for JNK1 substrate binding, whereas Phe287 plays a role for the precise alignment of active-site residues, which are important for transition-state stabilization^32^. This newly identified FXF-type motif is present in all MKPs, except that the residue at the first position in MKP5 is an equivalent hydrophobic leucine residue (see also Fig. 7f,g), suggesting that these two Phe residues would play a similar role in facilitating substrate recognition and catalysis, respectively. An important feature of MKP–JNK1 interactions is that MKP7 or MKP5 only interact with the F-site of JNK1. One possible explanation is that JNK1 needs to use the D-site to interact with JIP-1, a scaffold protein for JNK signalling^50^,^51^. The N-terminal JNK-binding domain of JIP-1 interacts with the D-site on JNK and this interaction is required for JIP-1-mediated enhancement of JNK activation^52^,^53^. In addition, JIP-1 can also associate with MKP7 via the C-terminal region of MKP7 (ref. 54). When MKP7 is bound to JIP-1, it reduces JNK activation, leading to reduced phosphorylation of the JNK target c-Jun. Thus, our biochemical and structural data allow us to present a model for the JNK1–JIP-1–MKP7 ternary complex and provide an important insight into the assembly and function of JNK signalling modules (Supplementary Fig. 6).

## Methods

### Protein preparation

The cDNAs of human MKP7 and MKP5 were kindly provided by Dr Mathijs Baens (University of Leuven) and Dr Eisuke Nishida (Kyoto University), respectively. The cDNAs of human ASK1, MKK4, MKK7 and JNK1 were kindly provided by Dr Zhenguo Wu (Hong Kong University of Science and Technology). The catalytic domains of MKP7 (MKP7-CD, residues 156–301) and MKP5 (MKP5-CD, 320–467) and the full-length MKP5 were cloned into the pET15b vector, resulting in the N-terminal His-fusion proteins. The KBD domains of MKP7 (MKP7-KBD, 5–138) and MKP5 (MKP5-KBD, 139–287), and the C-terminal truncation of MKP7 (MKP7ΔC304, 5–303) were cloned into pET21b vector for generation of C-terminal His-tagged proteins. The human full-length JNK1, MKK4, MKK7 and the kinase domain of ASK1 (659–951) were cloned into pGEX4T-2, pET15b and/or pET21b vectors to produce a GST- or His-tagged protein. Mutations of MKP7-CD, MKP5-CD and JNK1 were generated by overlap PCR procedure. All constructs were verified by DNA sequencing. All proteins, overexpressed in BL21(DE3) cells at 20 °C, were first purified over Ni-NTA (Qiagen) or GS4B (GE Healthcare) columns, and then by ion exchange and gel filtration chromatography (Source-15Q/15S and Superdex-200, GE Healthcare) at 4 °C. The double-phosphorylated JNK1 (phospho-JNK1) was generated by mixing JNK1 with upstream kinases MKK4, MKK7 and ASK1 in buffer containing 10 mM MgCl_2_ and 2 mM ATP, and further purified by gel filtration chromatography (Superdex-200, GE Healthcare) at 4 °C (ref. 55). Proteins were stored at −80 °C, and stocks for phosphatase assays were supplemented with glycerol to a final concentration of 20% (v/v). Protein concentrations were determined spectrophotometrically using theoretical molar extinction coefficients at 280 nm (ref. 56).

### Crystallography

The mixture of unphosphorylated JNK1 and MKP7-CD at 1:1 molar ratio was subjected to crystallization trials. Crystals were grown by the vapor-diffusion technique in hanging drops, and the drops were prepared by mixing equal volumes of protein with reservoir solution containing 0.1 M HEPES, pH 7.0, 14% PEG3350, 0.2 M MgCl_2_, 6% 1,6-Hexanediol and 0.005 M EDTA at 21 °C. Crystals were cryo-protected in reservoir solutions supplemented with 10% glycerol and then flash frozen in liquid nitrogen. The diffraction data sets were collected at beamline 17U at Shanghai Synchrotron Radiation Facility and processed with the HKL2000 package^57^. The crystals belong to space group *P*1 and comprise eight molecules per asymmetric unit (four complexes). Structure was solved by molecular replacement using Phaser^58^ with JNK1 (PDB 1UKH) and MKP5-CD (PDB 1ZZW) as the search models. Standard refinement was performed with programs PHENIX^59^ and Coot^60^. The crystal structure of unphosphorylated JNK1 in complex with the catalytic domain of MKP7 was refined to 2.4 Å resolution. Initial structural refinement was performed with NCS restraints, and after several rounds the restraints were removed from the calculations. The final *R*_work_ and *R*_free_ were 21.7 and 23.9%, respectively. The crystallographic asymmetric unit contains four JNK1–MKP7-CD complexes. The four complexes are nearly identical with an r.m.s.d.<1 Å for any complex pair in the asymmetric unit. Ramachandran analysis was carried out using PROCHECK^61^. Additional density at the active site of MKP7-CD was attributed to a chloride ion incorporated as a crystallizing agent, similar to those observed in the structures of MKP3-CD and MKP5-CD (refs 62, 63). The data collection and refinement statistics are summarized in Table 1. All structural representations in this paper were prepared with PYMOL (http://www.pymol.org).

### Phosphatase assays

The activities of MKP7 and MKP5 was assayed using phospho-JNK1 as substrate in the coupled enzyme system containing 50 mM MOPS, pH 7.0, 100 mM NaCl, 0.1 mM EDTA, 50 μM MESG and 0.1 mg ml^−1^ PNPase. This coupled assay uses PNPase and its chromogenic substrate MESG to monitor the production of inorganic phosphate^64^. The reactions were initiated by addition of 0.4 μM MKP7-CD and MKP7ΔC304 (or 0.1 μM MKP5 full-length and 0.15 μM MKP5-CD) for substrate phospho-JNK1. All experiments were carried out at 25 °C in 1.8 ml reaction mixtures, and the continuous absorbance changes were recorded with a PerkinElmer LAMBDA 45 spectrophotometer equipped with a magnetic stirrer in the cuvette holder. The quantification of inorganic phosphate produced was monitored at 360 nm with the extinction coefficient of 11,200 M^−1^ cm^−1^ (ref. 65). The initial rates were determined from the linear slope of the progress curves obtained. The activity of MKP7-CD or MKP5-CD mutants were assayed using *p*NPP or phospho-JNK1 as substrate. The phospho-JNK1 assay was performed as the same procedure mentioned above, and in the presence of wild type (as a control) or indicated mutants, and equal concentrations of phospho-JNK1. The *p*NPP assay was performed in the reaction mixture containg 50 mM MOPS, pH 7.0, 100 mM NaCl, 0.1 mM EDTA and 20 mM *p*NPP. The amount of the product *p*-nitrophenol was determined from the absorbance at 405 nm using a molar extinction coefficient of 18,000 M^−1^ cm^−1^ (ref. 31).

### Assays for protein–protein interaction

The interactions of JNK1 with the CD and KBD domains of MKP7 and MKP5 were examined by gel filtration analyses using a Superdex-200 10/300 column on an ÄKTA FPLC (GE Healthcare). The column was equilibrated with a buffer containing 10 mM HEPES, pH 7.5, 150 mM NaCl and 2 mM dithiothreitol, and calibrated with molecular mass standards. Samples of individual proteins and indicated mixtures (500 μl each) were loaded to the Superdex-200 column and then eluted at a flow rate of 0.5 ml min^−1^. Fractions of 0.5 ml each were collected, and aliquots of relevant fractions were subjected to SDS-polyacrylamide gel electrophoresis (PAGE) followed Coomassie Blue staining.

The interactions between various JNK1 mutants and MKP7-CD or MKP5-CD were assessed by GST-mediated pull-down assays at 4 °C. First, 0.5 ml GST-JNK1 proteins (6 μM) were loaded to 0.2 ml GS4B resin. The excess unbound JNK1 or other contaminants were removed by washing the column 5 times, each with 1.0 ml buffer containing 25 mM Tris-HCl, pH 8.0, 150 mM NaCl and 2 mM dithiothreitol. Then, 0.5 ml MKP7-CD or MKP5-CD (20 μM) was allowed to flow through the JNK1-bound column. After extensive washing, the bound proteins were eluted with 0.5 ml reduced glutathione (10 mM). The interactions of JNK1 with the CD and KBD domains of MKP7 and MKP5 were also examined by GST-mediated pull-down assays. The GST protein alone was used as a control. Aliquots of all eluates were subjected to SDS-PAGE, and proteins were visualized by Coomassie Blue staining. The uncropped gels are shown in Supplementary Fig. 7.

### Building JNK and MKP5 interaction model

The model of catalytic domain of MKP5 bound to JNK was constructed by superimposition of previous deposited structures of MKP5-CD (PDB 1ZZW) to the corresponding domains in the crystal structure of JNK1–MKP7-CD. The fractured loops in deposited structures were computationally generated using Modeller^66^. The program CHARMM22 (ref. 67) was then used to add hydrogen atoms, N- and C-terminal patches to the model. The model was then subjected to restrained energy minimization to optimize bonds and remove any nonbonded steric clashes. Refinement of the modelled complex was performed using NAMD2.9 package^68^ at 1 atm pressure and 300 K. The generated complex structure was solvated and neutralized in a box with TIP3P water at a minimum of 13 Å between the model and the wall of the box. The simulation was first set up with 1 fs time step under periodic boundary conditions. The particle mesh Ewald method was applied to model the electrostatics and the van der Waals interactions cutoff was set at 12 Å. The system was restrained for 5 ps minimization and 5 ps simulation, and followed by removing all the restraints and performing a minimization of 10 ps and an equilibration of 10 ns. Simulations were viewed using VMD^69^.

### Circular dichroism spectra

The experiments were performed on a Chirascan-plus circular dichroism Spectrometer (Applied Photophysics, Surrey, UK) using 0.1 mm quartz cuvette. The protein sample were analysed at a concentration of 0.5 mg ml^−1^. Data were collected over a wavelength range from 260 to 190 nm with 1 nm intervals at room temperature, three scans were averaged, and the baseline spetrum of solution buffer containing 10 mM HEPES (pH 7.5) and 150 mM NaCl was subtracted.

### Cell culture and transfections

pcDNA3.3-Myc-MKP7, pCMV5-3HA-JNK1 and pBOBI-HA-MKP7 were generated with standard molecular techniques. Mutants with amino acid substitution and truncation constructs were generated through PCR-based site-directed mutagenesis method using Pfu polymerase (Stratagene). The authenticities of all constructs were confirmed by sequencing (Invitrogen, China). HEK293T and HeLa cells (ATCC) were maintained in DMEM supplemented with 10% fetal bovine serum, 100 IU penicillin, 100 mg ml^−1^ streptomycin at 37 °C in a humidified incubator containing 5% CO_2_. Polyethylenimine (Polysciences, #23966) at a final concentration of 10 μM was used to transfect HEK293T cells. Total DNA for each plate was adjusted to the same amount by adding relevant blank vector. Lentiviruses for infection were packaged in HEK293T cells after transfection using Lipofectamine 2000 (Invitrogen, 11668-027). At 30 h post transfection, medium was collected for further infection.

### Coimmunoprecipitation and immunoblotting

Cells were lysed in a lysis buffer containing 20 mM Tris-HCl (pH 7.4), 150 mM NaCl, 0.5% NP-40, 1 mM EDTA, 2 mM Na_3_VO_4_, 25 mM NaF, 1 mM phenylmethanesulfonyl fluoride, 1 μg ml^−1^ leupeptin and 1 μg ml^−1^ aprotinin. Cell lysates were incubated with respective antibodies overnight at 4 °C. Protein aggregates resulting from the overnight incubation were removed by centrifugation, and protein A/G beads (Santa-Cruz Biotechnology, Dallas, TX, USA) were then added into the lysates and incubated for another 3 h. After spinning and washing for three times with the lysis buffer, the beads were mixed with 2 × SDS sample buffer, boiled and subjected to 15% SDS/PAGE. The samples were transferred to PVDF membranes (Millipore), and immunoblotted with indicated antibodies. Levels of total proteins and the levels of phosphorylation of proteins were analysed on separate gels. The uncropped blots are shown in Supplementary Fig. 7.

### Antibodies and drugs

Antibodies used in this study: mouse anti-HA (1:100 for immunoprecipitation; F-7) and anti-JNK1 (1:1,000 for immunoblotting; F-3) antibodies were purchased from Santa-Cruz Biotechnology. Anti-c-Myc Agarose Affinity Gel antibody produced in rabbit (1:200 for immunoprecipitation; A7470) was purchased from Sigma. Rabbit anti-HA-tag (1:1,000 for immunoblotting; #3724), anti-phospho-JNK-T183/Y185 (1:1,000 for immunoblotting; #4668) and mouse anti-Myc-tag (1:1,000 for immunoblotting; #2276) antibodies were purchased from Cell Signaling Technology. Etoposide (E1383) was purchased from Sigma.

### Apoptosis assay

HeLa cells were infected with lentiviruses expressing MKP7 or its mutants. At 36 h post infection, cells were irradiated with 25 J m^−2^ ultraviolet light and collected at 6 h after irradiation. Cells were then stained with the Annexin-V-APC/PI double-staining solution (BD Biosciences) and analysed with a flow cytometer (BD LSRFortessa). The percentages of apoptotic cells were quantified with FlowJo 7.6.1 software.

## Additional information

**Accession codes:** The coordinates and structure factors have been deposited in the Protein Data Bank with accession codes 4YR8 for the JNK1–MKP7-CD structure.

**How to cite this article:** Liu, X. *et al*. A conserved motif in JNK/p38-specific MAPK phosphatases as a determinant for JNK1 recognition and inactivation. *Nat. Commun.* 7:10879 doi: 10.1038/ncomms10879 (2016).

## Supplementary Material

Supplementary Information Supplementary Figures 1-7.

## Figures and Tables

**Table 1:** Data collection and refinement statistics.

	**JNK1–MKP7-CD**
Data collection*	
Space group	*P*1
Cell dimensions	
*a*, *b*, *c* (Å)	58.1, 74.8, 134.8
α, β, γ (°)	76.9, 84.3, 67.4
Resolution (Å)	40.00–2.40 (2.49–2.40)†
*R*_merge_	6.4 (60.9)
*I*/σ*I*	14.1 (1.9)
Completeness (%)	99.6 (99.9)
Redundancy	3.5 (3.4)
	
*Refinement*	
Resolution (Å)	38.02–2.40 (2.43–2.40)
No. of reflections	66889
*R*_work_/*R*_free_	21.7/23.9
No. atoms	
Protein	14637
Ligand/ion	4
Water	457
*B*-factors	
Protein	47.04
Ligand/ion	30
Water	37.28
R.m.s.d.	
Bond lengths (Å)	0.015
Bond angles (°)	1.564

^*^The data set was collected from a single crystal.

^†^Values in parentheses are for the highest resolution shell.
