# Supplementary material for: A conserved motif in JNK/p38-specific MAPK phosphatases as a determinant for JNK1 recognition and inactivation
Source: Nat Commun. 2016 Mar 18;7:10879. doi: 10.1038/ncomms10879 (PMC4802042; doi:10.1038/ncomms10879)
Supplement: Supplementary Information — Supplementary Figures 1-7. [file ncomms10879-s1.pdf]

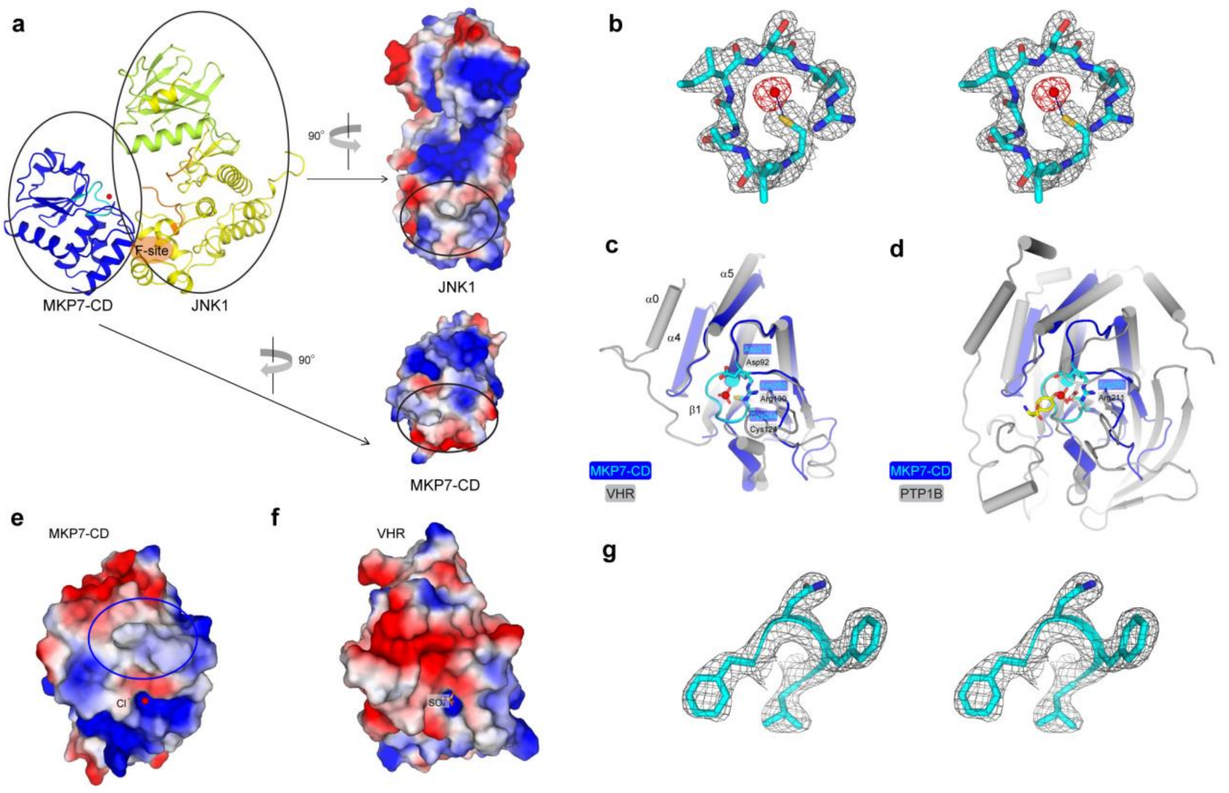

Supplementary Figure 1. Comparisons of MKP7-CD with VHR and PTP1B. (**a**) Electrostatic potential surfaces of JNK1-MKP7-CD complex. Interacting surfaces of MKP7-CD and JNK1 are shown in right and encircled in black. (**b**) The stereo image of the $2F_o - F_c$ omit map for the P-loop. (**c**) Superposition of MKP7-CD with VHR (PDB 1VHR). VHR is shown in gray, and its bound sulfate ion (orange stick) is well superimposed on the chloride ion identified at the active site of MKP7-CD. (**d**) Superposition of MKP7-CD with PTP1B (PDB 1PTY). PTP1B is colored in gray, and its bound phosphotyrosine is highlighted as yellow stick. Notably, the chloride ion in the MKP7-CD structure can be superimposed on the phosphate group of phosphotyrosine as well. (**e**) Electrostatic potential surfaces of MKP7-CD. Hydrophobic region of MKP7-CD that involved in binding JNK1 is encircled in blue. (**f**) Electrostatic potential surfaces of VHR. The orientation of the panel is almost identical to those of panel **e**. (**g**) The stereo image of the $2F_o - F_c$ omit map for the ^{285}FNFL^{288} segment.

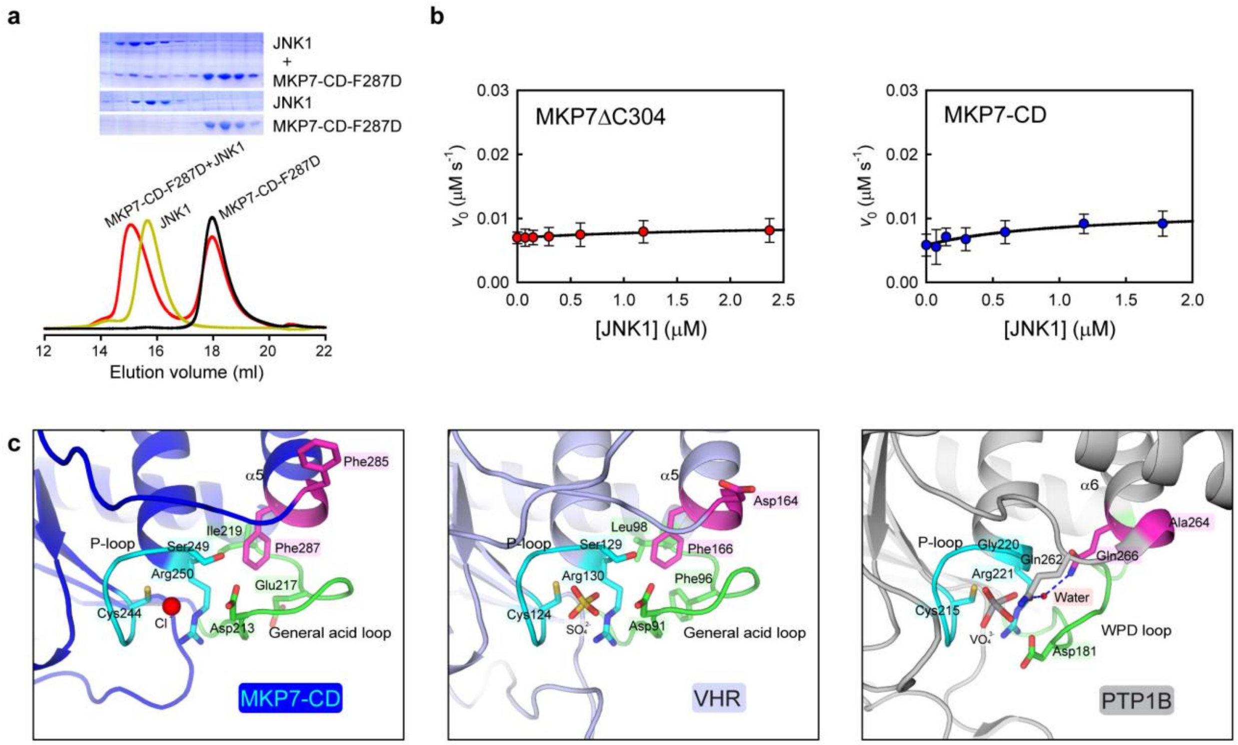

Supplementary Figure 2. Close-up view of the residue Phe287 in MKP7-CD structure. (**a**) Gel filtration analysis for interaction of JNK1 with MKP7-CD mutant F287D. When 3 molar equivalents of mutant F287D were mixed with 1 molar equivalent of JNK1, a significant amount of mutant F287D co-migrated with JNK1 to earlier fractions, indicating stable complex formation. (**b**) Hydrolysis of *p*NPP by MKP7 in the presence of the indicated concentrations of unphosphorylated JNK1. Each experiment was performed in replicate for at least three times. The error bars represent s.e.m.. (**c**) Comparison of MKP7-CD with PTP1B (PDB 3I80) and VHR (PDB 1VHR). VHR and PTP1B are shown in light blue and gray, respectively. The P-loop, general acid loop (WPD loop in PTP1B) and α5 (α6 in PTP1B) are highlighted as cyan, green and magenta, respectively, and critical residues are labeled. The water molecule found in PTP1B is shown in red. Blue dashed lines represent polar interactions.

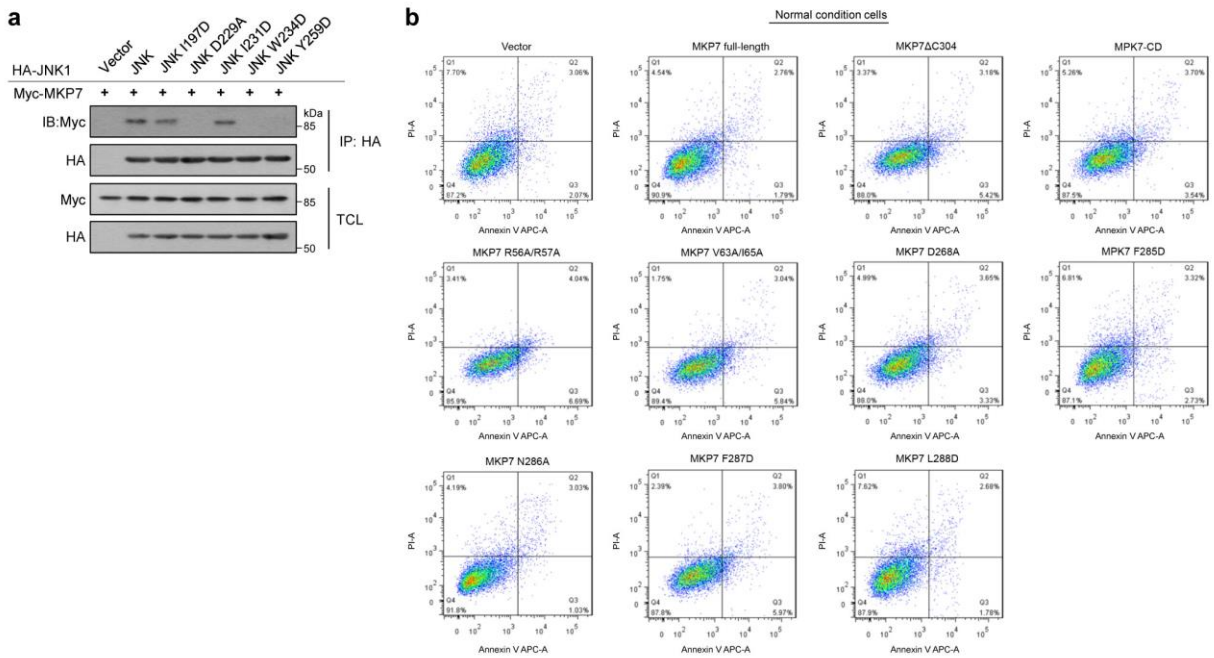

Supplementary Figure 3. F-site is crucial for mediating the MKP7 and JNK1 interaction. (**a**) F-site is required for JNK to interact with MKP7. HEK293T cells were co-transfected with MKP7 and JNK1 (wildtype or mutants as indicated, 1.0 μg). At 16 h post-transfection, cells were lysed. Whole cell extracts were then immunoprecipitated with antibody against HA for JNK1 followed by immunoblotting with antibodies indicated. IP, immunoprecipitation; TCL, total cell lysate. Shown is a typical result from three independent experiments. (**b**) Effect of MKP7 (wildtype or mutants) expression on apoptosis. HeLa cells were infected with lentiviruses expressing MKP7 and its mutants. At 36 h post-infection, cells were regularly cultured (control for the cells irradiated with 25 J m^{-2} UV) for 6 h and subjected to flow cytometry analysis. Apoptotic cells were determined by Annexin V-APC/PI staining.

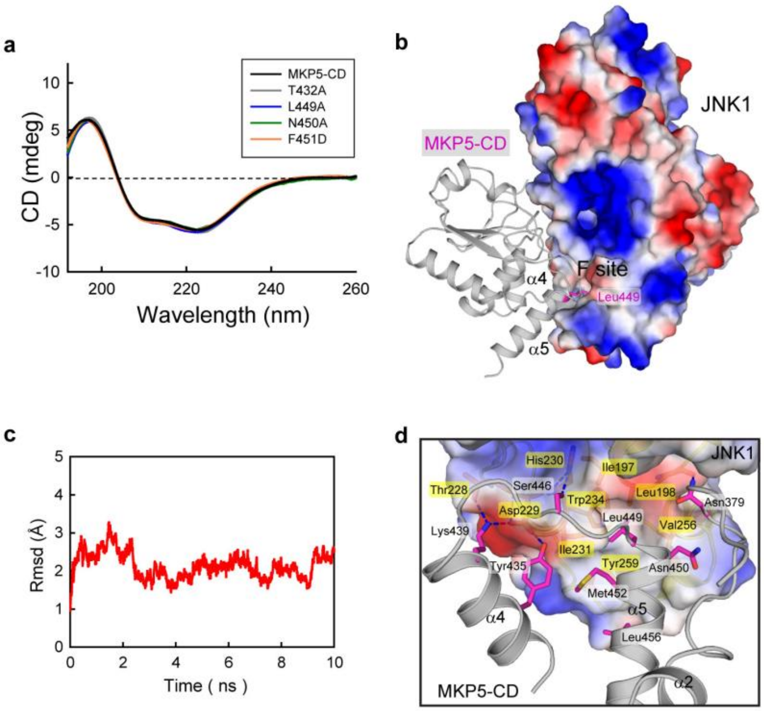

Supplementary Figure 4. A model of the JNK1-MKP5 complex. (**a**) Circular dichroism (CD) spectra for MKP5 wildtype and mutants. Measurements were averaged for three scans. (**b**) Results of simulations of JNK1-MKP5-CD complex. The MKP5-CD is colored in gray, and the JNK1 is shown in surface representation colored according to the electrostatic potential (positive, blue; negative, red). (**c**) Total backbone root mean square deviation (rmsd) for JNK1-MKP5-CD relative to the starting structure in simulation. (**d**) Contacts between MKP5-CD and JNK1. Residues of MKP5-CD and JNK1 are highlighted as pink and orange sticks, respectively. Blue dashed lines represent polar interactions.

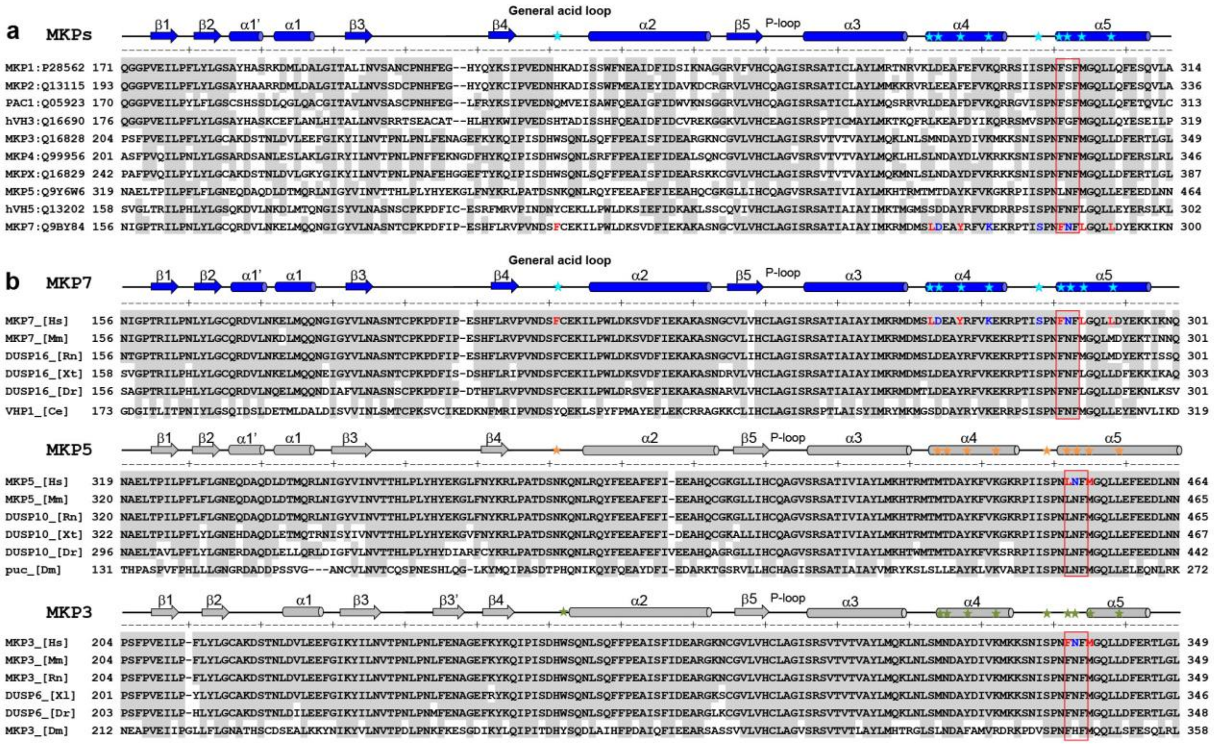

Supplementary Figure 5. Sequence alignment of MKPs generated by ClustalW. (**a**) Sequence alignment of different MKPs in human. Residues of MKP7-CD involved in JNK1 recognition are indicated by cyan asterisks. The secondary structure assignments of MKP7-CD are shown above sequences. The FXF-motifs are boxed. (**b**) Sequence alignment of MKP7-CD, MKP5-CD and MKP3-CD among different species, respectively. The secondary structure assignments of MKP7-CD, MKP5-CD and MKP3-CD are shown above sequences, respectively. *Hs, Homo sapiens; Mm, Mus musculus; Rn, Rattus norvegicus; Xt, Xenopus tropicalis; Xl, Xenopus laevis; Dr, Danio rerio; Ce, Caenorhabditis elegans; Dm, Drosophila melanogaster*. The Uniprot identifiers for MKP7 on different species are Q9BY84, Q920R2, D4A3W6, A1A5G3, Q6NXD7 and Q10038, respectively. The Uniprot identifiers for MKP5 on different species are Q9Y6W6, Q9ESS0, D3ZBG7, F6WGV1, F1QIT6 and Q9VHV8, respectively. The Uniprot identifiers for MKP3 on different species are Q16828, Q9DBB1, Q64346, Q91663, Q7T2L8 and Q9VVW5, respectively.

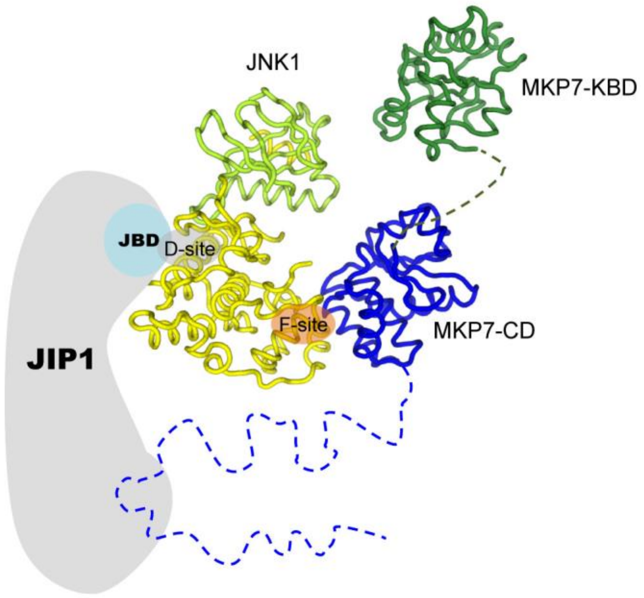

Supplementary Figure 6. A model of the JNK1-JIP1-MKP7 ternary complex. JIP-1 is shown in gray, and the color schemes for JNK1 and MKP7 are the same as that in Fig. 2a. JIP-1 specifically binds the MKP7 via a region independent of its JNK binding domain (JBD); MKP7 interacts with JNK1 and JIP1 through catalytic domain and C-terminal region, respectively; and JNK1 contacts with MKP7-CD through F-site, instead JIP-1 binds the D-site of JNK1. Through the specific binding of JNK1-JIP1-MKP7 ternary complex, MKP7 dephosphorylates JNK1 effectively.

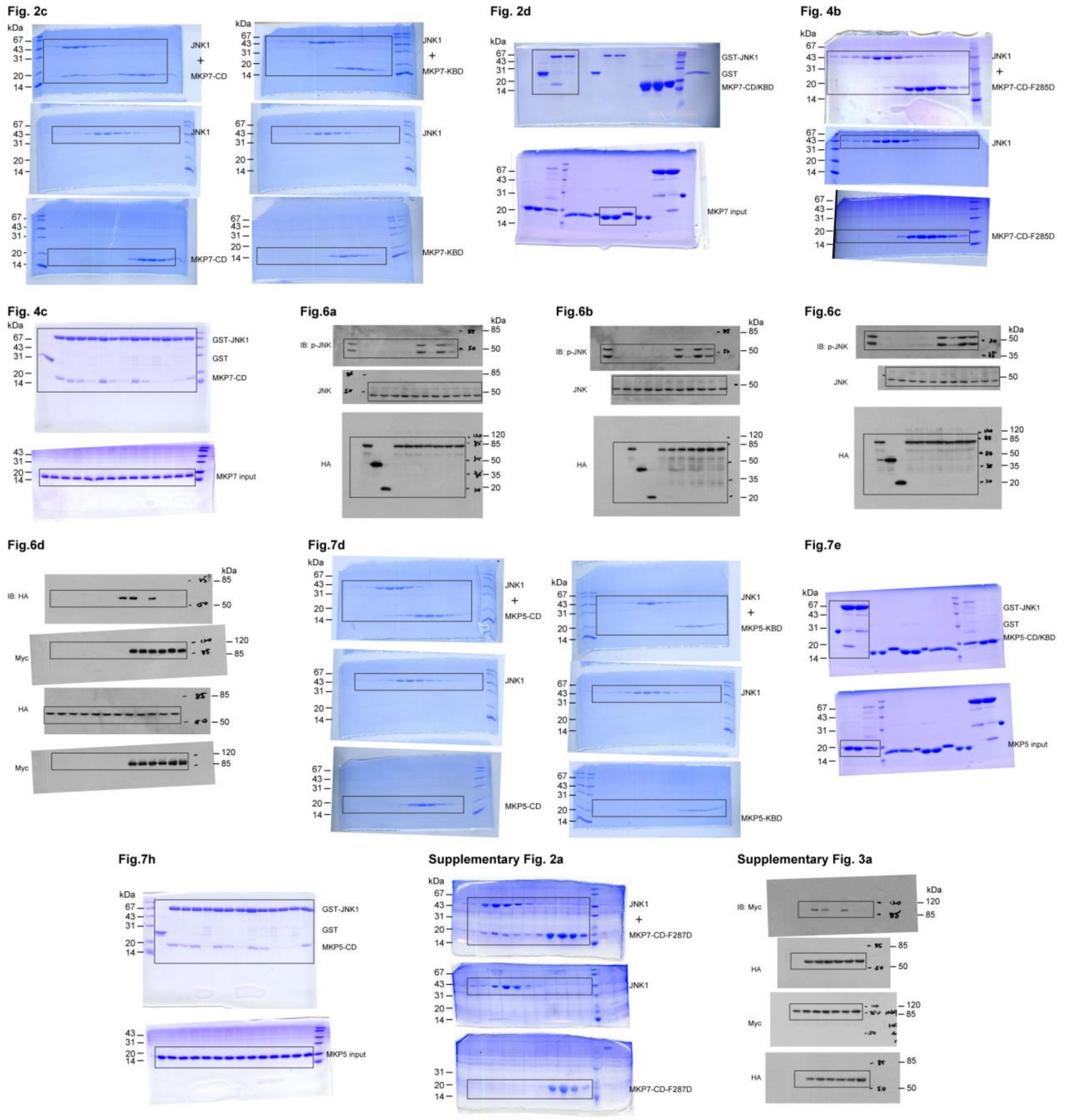

Supplementary Figure 7. Scans of uncropped blots and gels of the figures. Cropped regions are indicated with rectangles as appropriate.
